# Exploring Applications of Radiomics in Magnetic Resonance Imaging of Head and Neck Cancer: A Systematic Review

**DOI:** 10.3389/fonc.2018.00131

**Published:** 2018-05-14

**Authors:** Amit Jethanandani, Timothy A. Lin, Stefania Volpe, Hesham Elhalawani, Abdallah S. R. Mohamed, Pei Yang, Clifton D. Fuller

**Affiliations:** ^1^Department of Radiation Oncology, The University of Texas MD Anderson Cancer Center, Houston, TX, United States; ^2^College of Medicine, The University of Tennessee Health Science Center, Memphis, TN, United States; ^3^Baylor College of Medicine, Houston, TX, United States; ^4^Department of Oncology and Hemato-Oncology, University of Milan, Milan, Italy; ^5^Department of Clinical Oncology and Nuclear Medicine, Faculty of Medicine, University of Alexandria, Alexandria, Egypt; ^6^Graduate School of Biomedical Sciences, The University of Texas Health Science Center, Houston, TX, United States; ^7^Hunan Cancer Hospital, Department of Head and Neck Radiation Oncology, Changsha, China

**Keywords:** radiomics, magnetic resonance imaging, MRI, texture analysis, head and neck, radiation oncology

## Abstract

**Background:**

Radiomics has been widely investigated for non-invasive acquisition of quantitative textural information from anatomic structures. While the vast majority of radiomic analysis is performed on images obtained from computed tomography, magnetic resonance imaging (MRI)-based radiomics has generated increased attention. In head and neck cancer (HNC), however, attempts to perform consistent investigations are sparse, and it is unclear whether the resulting textural features can be reproduced. To address this unmet need, we systematically reviewed the quality of existing MRI radiomics research in HNC.

**Methods:**

Literature search was conducted in accordance with guidelines established by Preferred Reporting Items for Systematic Reviews and Meta-Analyses. Electronic databases were examined from January 1990 through November 2017 for common radiomic keywords. Eligible completed studies were then scored using a standardized checklist that we developed from Enhancing the Quality and Transparency of Health Research guidelines for reporting machine-learning predictive model specifications and results in biomedical research, defined by Luo et al. (1). Descriptive statistics of checklist scores were populated, and a subgroup analysis of methodology items alone was conducted in comparison to overall scores.

**Results:**

Sixteen completed studies and four ongoing trials were selected for inclusion. Of the completed studies, the nasopharynx was the most common site of study (37.5%). MRI modalities varied with only four of the completed studies (25%) extracting radiomic features from a single sequence. Study sample sizes ranged between 13 and 118 patients (median of 40), and final radiomic signatures ranged from 2 to 279 features. Analyzed endpoints included either segmentation or histopathological classification parameters (44%) or prognostic and predictive biomarkers (56%). Liu et al. (2) addressed the highest number of our checklist items (total score: 48), and a subgroup analysis of methodology checklist items alone did not demonstrate any difference in scoring trends between studies [Spearman’s ρ = 0.94 (*p* < 0.0001)].

**Conclusion:**

Although MRI radiomic applications demonstrate predictive potential in analyzing diverse HNC outcomes, methodological variances preclude accurate and collective interpretation of data.

## Introduction

### Rationale

Tumor characterization remains a major obstacle in the treatment of HNC patients (3, 4). Structural heterogeneity may represent underlying differences in tumor biology, which often cannot be explained by clinical data alone (5–8). Radiomics, the quantitative evaluation of anatomic structures from diagnostic imaging modalities, could possibly mitigate this variance (5, 6, 9). By describing morphological parameters and textural features from voxel elements, radiomics has the potential to examine tumors entirely (10–13).

Although multiple studies have applied radiomic analyses in HNC patients, computed tomography (CT) is the imaging modality most frequently investigated (14–26). This preference is due, in part, to the relative ease of data extraction and interpretation: Textural features can be derived from CT signal intensities (SIs) because their units of measurement, Hounsfield units (HUs), directly represent tissue radiodensity. Thus, SI gradients contain information about structural properties, which could then be translated into clinically meaningful data (9).

Computed tomography affords yet another advantage in that its imaging performance tends to be standardized across scanners and vendors (9). However, CT acquisition parameters can still influence the appearance of radiomic features (27). In non-small cell lung cancer (NSCLC), Mackin et al. (27) designed a radiomics-specific CT phantom to test inter-scanner variability. Mean CT number, reflected in HU, approximated the same variability between extracted tumor features from the scans themselves (27). Although extraction of features with discriminative ability from multiple scanners is promising, research is lacking in their application and robustness. Likewise, variances in reconstruction algorithms and image noise represent barriers to the accuracy of extracted features (9).

Similarly, radiomic studies based on magnetic resonance imaging (MRI) also face derivational challenges intrinsic to the technology. Not only are scanner parameters obstacles to reproducibility of features, but images themselves may reflect multiple tissue properties with specific acquisition characteristics (28). For instance, MRI SIs depends on pulse sequences, relaxation times, as well as a host of other acquisition-related processes; thus, seamless integration of radiomic analyses requires substantive effort (28).

When conducted appropriately, however, such studies can potentially provide a breadth of information superior to extrapolated values from CT radiomic features, as multiple physical properties of a voxel can be extracted *via* distinct sequence acquisition processes (e.g., spin–spin, proton density) and could be leveraged even further using novel techniques for simultaneous voxel characterization (e.g., MR fingerprinting) (29).

For example, MRI radiomics could potentially describe distinct patterns in tumor physiology: phenotypic categories from diffusion-weighted imaging (DWI) and dynamic contrast-enhanced (DCE) MRI have successfully predicted prognostic status in breast cancer patients (30). In addition, radiomic features derived from T1-weighted MRI reliably categorized molecular subtypes of breast tumors (31). For cases of glioblastoma (GBM), MRI radiomic profiles outperformed clinical and radiologic risk models in stratification of survival (32). Radiomic features have also successfully classified prostate tumors by Gleason scores (33, 34).

### Objectives and Research Question

To the best of our knowledge, MRI radiomic applications in HNC have yet to be systematically summarized and reviewed in the clinical literature. In this effort, we assessed the quality of existing research: We comprehensively described MRI radiomic studies specific to the head and neck sub-site, with an intentional focus on study design. We compare and contrast the studies with a checklist based on Luo et al. (1) Enhancing the Quality and Transparency of Health Research (EQUATOR) methodology reporting guidelines. Subsequently, we discuss ongoing clinical trials and suggest future directions for MRI radiomic applications in HNC. The purpose of this systematic review is to assess the level of evidence and gauge the applicability of MRI radiomics in HNC.

## Methods

### Study Design and Systematic Review Protocol

**Figure 1:**
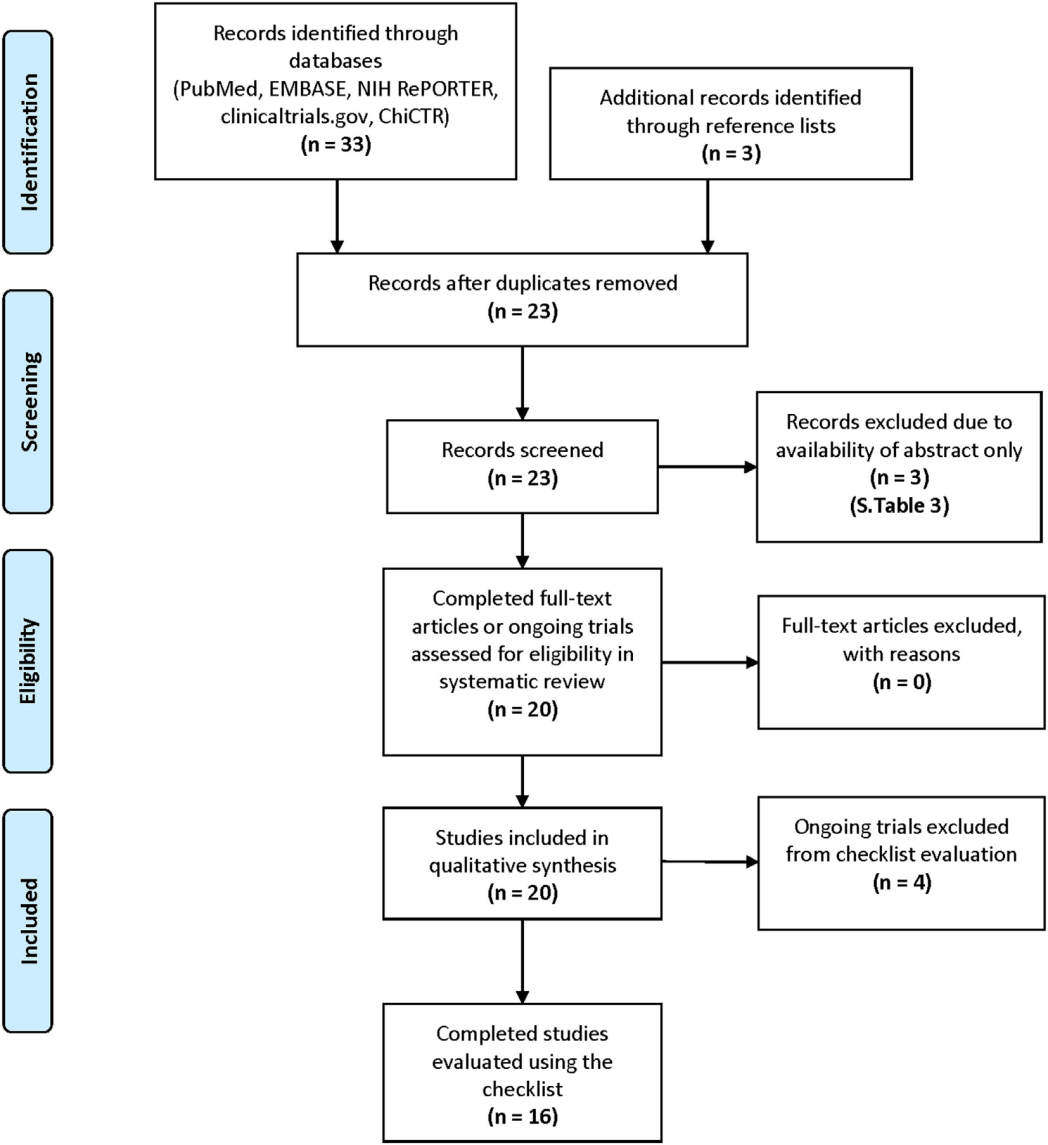
Study methodology and search strategy *via* Preferred Reporting Items for Systematic Reviews and Meta-Analyses guidelines (35).

Study methodology followed outlines established by Preferred Reporting Items for Systematic Reviews and Meta-Analyses (Figure 1).

#### Eligibility Criteria

Full-text, original manuscripts, published in English, accepted for publication, and available online or in-print were evaluated. For inclusion, study populations consisted of patients diagnosed with HNC. All other cancer populations were excluded. Interventions included investigations of MRI radiomic features, where MRI was the primary imaging modality implemented. Studies exclusively researching first-order MRI features were excluded as they did not accurately represent the scope of typical MRI radiomic applications in HNC. Regarding outcomes, studies were included if they investigated segmentation accuracy, histopathological classification parameters, or prognostic and predictive biomarkers. Study design could be observational (e.g., prospective cohort, retrospective cohort, and case–control) or a clinical trial (e.g., randomized controlled trial).

#### Study Search Strategy and Process

Electronic databases (National Center for Biotechnology Information PubMed, Elsevier EMBASE, National Institute of Health Research Portfolio Online Reporting Tool, ClinicalTrials.gov, and the Chinese Clinical Trial Registry) were searched from January 1990 through November 2017. Keywords and search strategy are described in our supplementary material (Table S5). For each included manuscript, reference lists were searched for additional eligible studies. Study search was completed by three authors independently (Amit Jethanandani, Timothy A. Lin, and Stefania Volpe), reviewing manuscripts in a stepwise method: By title alone, followed by abstract, then full-text. Search results were imported into individual spreadsheets using JMP Pro software version 12.1.0 (SAS Institute Inc., Cary, NC, USA). Discrepancies between results were discussed at team meetings, moderated by a fourth author (Hesham Elhalawani). Study search and selection were completed on November 13, 2017.

### Data Sources, Study Sections, and Data Extraction

Selected studies consisted of completed research and ongoing trials. Once a final list was established, data extraction was completed independently by two authors (Amit Jethanandani and Timothy A. Lin) then assessed for quality by a third author (Hesham Elhalawani). Information was extracted into JMP Pro spreadsheets and included the following data: Manuscript title; authors; publication date; number of patients; head and neck sub-site; MRI modality and/or sequence used for radiomics analysis; region of interest (ROI) segmentation method; image pre-processing; feature extraction software; analyzed endpoint; statistical findings: radiomic model performance; conclusions; search terms and databases used to identify selected studies. Completed studies were stratified based on endpoints evaluated: Segmentation or histopathological classification vs. prognostic or predictive measures. Synthesis of data into a final spreadsheet was accomplished at team meetings among three authors (Amit Jethanandani, Timothy A. Lin, and Hesham Elhalawani).

#### Checklist Construction

A qualitative scoring method was developed for independent evaluation of completed studies. This system was adapted from Luo et al. (1) EQUATOR methodology reporting guidelines, which represent criteria outlined by a multidisciplinary panel of 11 clinicians, machine-learning specialists, and expert statisticians. The guidelines aimed to achieve two main objectives: (1) establish a list of key reporting items and (2) design a standardized, stepwise approach for generation of predictive models. The Delphi method was leveraged to iteratively narrow a list of included topics, discussed over e-mail between the panel members, to the final guidelines.

The guidelines were categorized by manuscript section for each reporting item: Title and abstract, introduction, methods, results, and discussion. Within these categories, reporting items were grouped by subsection. For example, the methods section contained the following groups: “Describe the setting,” “define the prediction problem,” “prepare data for model building,” “build the predictive model,” and “report the final model and performance.” Our checklist mirrored this organization, with a few exceptions: Within the “build the predictive model” subsection, we further defined “data (feature) pre-processing” and “basic statistics of the dataset.” Data pre-processing refers to data cleaning, data transformation, outlier removal, criteria for outlier removal, and handling of missing values. Basic statistics included items clarifying whether the model reflected the chosen classification or regression problem, the validation strategy, validation metrics, and the starting time for validation data collection. For organization of reporting items, a blank checklist is provided in our supplementary data section (Table S1 in Supplementary Material).

Each mandatory checklist item was categorized into a yes/no binary variable, which indicated whether the study appropriately addressed the corresponding criteria. The checklist was designed by one author (Timothy A. Lin) and subsequently revised by two authors (Amit Jethanandani and Hesham Elhalawani). Each completed study was scored individually by two authors (Amit Jethanandani and Timothy A. Lin). After all completed studies were scored, a group of three authors (Amit Jethanandani, Timothy A. Lin, and Hesham Elhalawani) met together to resolve discrepancies. There were 55 total checklist items, with two items containing sub-scores, representing a maximum overall score of 58 points. Once total checklist scores [total score (TS)] were finalized, methodology scores (MS) alone were generated for each completed study.

### Data Analysis

Descriptive statistics for all included studies were populated and reviewed. For completed studies, TS and MS were tabulated in JMP Pro software. In addition, a subgroup analysis comparing collinearity of MS to TS was conducted using Spearman’s ρ. Subgroup analysis was completed using the same JMP Pro software mentioned earlier.

## Results

### Study Selection and Characteristics

Sixteen completed (2, 36–50) and four ongoing studies (51–54) were selected for inclusion. For completed studies, online or print publication dates ranged between May 2013 and October 2017. The selected studies could be retrieved from PubMed, and the most successful search term was “MRI texture analysis” (50% discovered with this keyword alone).

### Synthesized Findings of Completed Studies

Patient sample sizes ranged between 13 and 118 patients with a median of 40 patients (Table 1). Head and neck sub-sites were diverse, including tumor volumes as well as normal anatomic structures. Of studies extracting radiomic features from tumor volumes, nasopharyngeal cancer (NPC) studies (37.5%) were the most common. Investigations of radiotherapy (RT)-related toxicities in normal tissue composed a small sample of the cohort (12.5%). Specific sub-sites were unknown for two studies (12.5%).

**Table 1:** Magnetic resonance imaging (MRI) radiomics in HNC: completed studies

Article title	Article authors	Publication date	Number of patients	Head and neck sub-site	MRI modality and/or sequence used for radiomics analysis	Region of interest (ROI) segmentation method	Image pre-processing: yes/no	Feature extraction software	Analyzed endpoint	Statistical findings: radiomic model performance	Conclusions	Successful search terms used [1 = Radiomic(s), 2 = MRI texture analysis, 3 = texture analysis, 4 = head and neck, 5 = magnetic resonance imaging texture analysis]	Databases [1 = PubMed, 2 = EMBASE, 3 =NIH, 4 = ClinicalTrials.Gov, 5 = Chinese Clinical Trial Registry (ChiCTR)]
Studies on radiomics for segmentation and histopathological classification													
MRI texture analysis reflects histopathology parameters in thyroid cancer—a first preliminary study	Meyer HJ, Schob S, Hohn AK, Surov A	10/6/2017 (electronic publication, ePub); 12/2017 (Print)	13	Thyroid	T1-weighted turbo spin echo (TSE); T2-weighted TSE	Not specified	Yes	MaZda	Histopathological classification	279 texture features were analyzed for univariate association with histological parameters using a Spearman’s correlation coefficient	Several significant correlations were identified between texture features and histopathology	2	1

Multi-institutional validation of a novel textural analysis tool for preoperative stratification of suspected thyroid tumors on diffusion-weighted MRI	Brown AM, Nagala S, McLean Ma, Lu Y, Scoffings D, Apte A, Gonen M, Stambuk HE, Shaha AR, Tuttle RM, Deasy JO, Priest AN, Jani P, Shukla-Dave A, Griffiths J	5/20/2015 (ePub); 4/2016 (Print)	42 (training=24, validation=18)	Thyroid	Diffusion-weighted imaging (DWI)	Manual	Yes	MaZda	Histopathological classification	A linear discriminant analysis (LDA) model of the top 21-ranking MaZda textural features classified 89/94 ROIs with 92% sensitivity and 96% specificity [AUC: 0.97, 95% confidence interval (CI): 0.92–1.0]. In a test set of 18 cases, the model’s sensitivity was 89% (95% CI: 65–99%) and its specificity was 97% (95% CI: 74–100%)	Texture analysis is sensitive and specific for stratification of thyroid nodules	2	1

MRI texture analysis predicts p53 status in head and neck squamous cell carcinoma	Dang M, Lysack JT, Wu T, Matthews TW, Chandarana SP, Brockton NT, Bose P, Bansal G, Cheng H, Mitchell JR, Dort JC	9/25/2014 (ePub); 1/2015 (Print)	16	Oropharynx	Contrast-enhanced T1-weighted FSE; T2-weighted fast spin echo (FSE) with fat saturation; DWI	Manual	Yes	2D Fast Time-Frequency Transform Tool	Histopathological classification	A model of seven significant variables (determined using a subset-size forward selection algorithm and isolation of high-classification percentage variables) correctly classified 81.3% of tumors (κ: 0.625, *p* < 0.05)	A radiomic model containing variables with high classification performance could predict p53 status in oropharyngeal cancer patients	2	1

Texture-based analysis of 100 MR examinations of head and neck tumors—is it possible to discriminate between benign and malignant masses in a multicenter trial?	Fruehwald-Pallamar J, Hesselink JR, Mafee MF, Holzer-Fruehwald L, Czerny C, Mayerhoefer ME	9/30/2015 (ePub); 2/2016 (Print)	100	Head and neck benign (cysts = 8, inflammatory masses = 5, parotid = 9, glomus = 9, vascular malformation = 5, schwannoma = 4, other = 6) and malignant (squamous cell carcinoma = 31, lymphoma = 8, adenoid cystic = 5, adeno = 4, other = 6) tumors	Various	Manual and autosegmentation	No	MaZda	Histopathological classification	LDA models based off subsets of previously-identified, significant texture features demonstrated differences on STIR (61.29–80.65%) and T2-weighted images (T2-TSE: 81.82–100%, T2-TSE with fat suppresion: 71.74–78.26%) for 2D evaluation and on contrast-enhanced T1-TSE with fat saturation (58.54–85.37%) for 3D evaluation. Secondary analysis of subgroups by Tesla strength was also conducted	Texture analysis is not practical for differentiation of tumors using different magnetic resonance (MR) protocols on different MR scanners	2	1

Automated segmentation of the parotid gland based on atlas registration and machine learning: a longitudinal MRI study in head-and-neck radiation therapy	Yang X, Wu N, Cheng G, Zhou Z, Yu DS, Beitler JJ, Curran WJ, Liu T	10/13/2014 (ePub); 12/2014 (Print)	15	Head and neck (oropharynx and larynx but other sites not specified)	Contrast-enhanced T1-weighted; Contrast-enhanced T2-weighted	Manual and autosegmentation	Yes	Not specified	Segmentation accuracy	A three-step autosegmentation method leveraging, as a component, a trained kernel-based support vector machine (SVM) model successfully differentiated 100% of parotid volumes where the average percentage of volume differences between the proposed method and manual physician contours were 7.98% (left parotid) and 8.12% (right parotid). Average Dice volume overlap: 91.1 ± 1.6% (left) and 90.5 ± 2.4% (right). Significant differences in volume reductions were found between 3-month and 1-year follow-up examinations (*p* = 0.19) and between 6-month and 1-year follow-up examinations (*p* = 0.14)	An autosegmentation method leveraging SVM models could accurately segment parotid glands when compared with manual review by trained experts	2	1

Texture-based and diffusion-weighted discrimination of parotid gland lesions on MR images at 3.0 Tesla	Fruehwald-Pallamar J, Czerny C, Holzer-Fruehwald L, Nemec SF, Mueller-Mang C, Weber M, Mayerhoefer ME	5/23/2013 (ePub); 11/2013 (Print)	38	Parotid masses	Contrast-enhanced T1-weighted TSE; T1-weighted TSE; T1-weighted with fat suppression; Short Tau Inversion Recovery (STIR)	Manual and autosegmentation	Yes	MaZda	Histopathological classification	LDA models based off subsets of previously-identified, significant texture features was leveraged to determine differences between benign and malignant parotid masses or pleomorphic adenomas and Warthin tumors on multiple imaging modalities. Contrast-enhanced T1-weighted features correctly classified 81.8–84.5% of benign-malignant masses. Whereas, the same models applied to STIR imaging was poorer in distinguishing benign-malignant masses (73.5–78.4%) and pleomorphic adenomas-Warthin tumors (50–59%)	Contrast-enhanced T1-weighted features contained the most predictive textural information for distinguishing benign and malignant parotid masses. STIR images contained the least relevant textural information	2	1

MRI-based texture analysis to differentiate sinonasal squamous cell carcinoma from inverted papilloma	Ramkumar S, Ranjbar S, Ning S, Lal D, Zwart CM, Wood CP, Weindling SM, Wu T, Mitchell JR, Li J, Hoxworth JM	3/2/2017 (ePub); 5/2017 (Print)	46 (training=33, validation=13)	Sinonasal	Contrast-enhanced T1-weighted with fat suppression; T1-weighted; T2-weighted with fat suppression	Manual and autosegmentation	Yes	Python	Histopathological classification	The classification model, developed using five texture algorithms, demonstrated 90.9% accuracy in the training set and 84.6% accuracy in the validation set (*p* = 0.537). With both sets included, model accuracy (89.1%) outperformed neuroradiologists’ ROI review (56.5%, *p* = 0.0004). This was not significantly different from neuroradiologist review of tumors (73.9%, *p* = 0.060) or entire images (87%, *p* = 0.748)	Machine-learning accuracy of texture analysis algorithms outperformed neuroradiologists’ region of interest (ROI) review in classification of sinonasal carcinomas vs. inverted papillomas; however, its accuracy was not significantly different from neuroradiologists’ review of tumors or entire images	2	1

Studies on radiomics for prognostic and predictive biomarkers													
Exploration and validation of radiomics signature as an independent prognostic biomarker in stage III-IVb nasopharyngeal carcinoma	Ouyang FS, Guo B, Zhang B, Dong Y, Zhang L, Mo X, Huang W, Zhang S, Hu Q	9/26/2017 (ePub); 8/24/2017 (Print)	100 (training=70, validation=30)	Nasopharynx	Contrast-enhanced T1-weighted; T2-weighted	Manual	Yes	Matlab	PFS (Progression free survival)	In both the discovery and validation sets, a radiomic signature—using features selected via least absolute shrinkage and selection operator (Lasso) regression—successfully stratified patients by PFS risk category (HR: 5.14, *p* < 0.001; HR: 7.28, *p* = 0.015) while other identified clinical-pathologic risk factors for PFS were not significant (all *p* for HR > 0.05).	A radiomic signature based off pre-treatment MRI scans could predict PFS risk category and improve clinical decision-making	1	1

Advanced nasopharyngeal carcinoma: pre-treatment prediction of progression based on multi-parametric MRI radiomics	Zhang B, Ouyang FS, Gu D, Dong Y, Zhang L, Mo X, Huang W, Zhang S	9/22/2017 (ePub); 8/2/2017 (Print)	113 (training=80, validation=33)	Nasopharynx	Contrast-enhanced T1-weighted; T2-weighted	Manual	No	Matlab	Progression (Dichotomized to Yes and No categories)	Similar to the above strategy, radiomic features were selected using least absolute shrinkage and a Lasso method for significant association with progression. In both the training and validation cohort, the resulting radiomic-based model optimally performed when derived from combined contrast-enhanced T1-weighted and T2-weighted imaging (training: AUC: 0.896, 95% CI: 0.815–0.956; validation: 0.823, 95% CI: 0.645–1.00)	A radiomic model based on contrast-enhanced T1 and T2 features outperformed a model based on either MRI modality alone in its ability to predict progression in advanced nasopharyngeal cancer (NPC)	1	1

Radiomic machine-learning classifiers for prognostic biomarkers of advanced nasopharyngeal carcinoma	Zhang B, He X, Ouyang FS, Gu D, Dong Y, Zhang L, Mo X, Huang W, Tian J, Zhang S	6/10/2017 (ePub); 9/10/2017 (Print)	110 (training=70, validation=40)	Nasopharynx	Contrast-enhanced T1-weighted; T2-weighted	Manual	Yes	Matlab	Prognostic performance of predicting local or distant treatment failure	Of the six feature selection and nine classification methods examined, the best predictive model utilized a combination Random Forest method (AUC: 0.8464 ± 0.0069; test error, 0.3135 ± 0.0088)	Radiomics models utilizing random forest methods demonstrated the highest prognostic performance compared with other machine-learning classification schemes, suggesting its utility in enhancing applications of radiomics in precision oncology	1	1

Radiomics features of multi-parametric MRI as novel prognostic factors in advanced nasopharyngeal carcinoma	Zhang B, Tian J, Dong D, Gu D, Dong Y, Zhang L, Lian Z, Liu J, Luo X, Pei S, Mo X, Huang W, Ouyang FS, Guo B, Liang L, Chen W, Liang C, Zhang S	3/9/2017 (ePub); 8/1/2017 (Print)	118 (training=88, validation=30)	Nasopharynx	Contrast-enhanced T1-weighted; T2-weighted	Manual	No	Matlab	PFS	Radiomic features were selected using least absolute shrinkage and a Lasso method for PFS nomograms. Radiomic signatures were significantly associated with PFS, with signatures derived from joint contrast-enhanced T1-weighted and T2-weighted images (Training C-index: 0.758, 95% CI: 0.661–0.856; Validation C-index: 0.737, 95% CI: 0.549–0.924). Outperforming signatures from either modality alone. When combined with clinical characteristics, the radiomics signature outperformed clinical characteristics alone in predicting PFS in advanced NPC (C-index, 0.776 vs. 0.649; *p* < 1.60 × 10^−7^)	Multiparametric MRI-based radiomic nomograms demonstrate prognostic ability in predicting progression in NPC patients	1	1

Texture analysis on parametric maps derived from dynamic contrast-enhanced magnetic resonance imaging in head and neck cancer	Jansen JF, Lu Y, Gupta G, Lee NY, Stambuk HE, Mazaheri Y, Deasy JO, Shukla-Dave A	1/28/2016 (Print)	19	Oropharynx	Dynamic contrast-enhanced (DCE)	Manual	No	Matlab	Treatment response	Texture analysis on parametric DCE-MRI maps revealed energy of ve was higher in intra-treatment vs. pre-treatment scans (*p* < 0.04)	Pharmokinetic models performed on DCE images, producing ktrans and ve maps, were unable to predict treatment response. However, imaging biomarker E of ve was significantly higher in intra-treatment scans, vs. pre-treatment scans, suggesting a possible change in heterogeneity. The study ultimately conlcudes chemoradiation treatment reduces tumor heterogeneity in this patient cohort	2	1

Use of texture analysis based on contrast-enhanced MRI to predict treatment response to chemoradiotherapy in nasopharyngeal carcinoma	Liu J, Mao Y, Li Z, Zhang D, Zhang Z, Hao S, Li B	1/18/2016 (ePub); 8/2016 (Print)	53 (training=42, validation=11)	Nasopharynx	Contrast-enhanced T1-weighted; T2-weighted; DWI; STIR TSE	Manual	Yes	Matlab	Treatment response	Three parameter sets of texture features derived from their respective imaging modalities were iteratively curated using multiple selection (e.g., the dynamic range metric) and classification methods (e.g., LDA). All three (T1: 0.952/0.939, T2: 0.904/0.905, DWI: 0.881/0.929) demonstrated an ability to predict treatment response, with supervised learning models using features from T1-weighted models exhibiting the highest classification performance vs. T2-weighted [artificial neural network (ANN): *p* = 0.043, k-nearest neighbors (k-NN): *p* = 0.033] or DWI (ANN: *p* = 0.032, k-NN: *p* = 0.014)	Radiomic models exhibit an ability to predict treatment response in NPC patients	2	1

Characterization of cervical lymph-nodes using a multi-parametric and multi-modal approach for an early prediction of tumor response to chemo-radiotherapy	Scalco E, Marzi S, Sanguineti G, Vidiri A, Rizzo G	9/14/2016 (ePub); 12/2016 (Print)	30	Head and neck (sites not specified)	T2-weighted; DWI; computed tomography (CT)	Manual	Yes	Python	Treatment response	Pre-treatment features outperformed mid-chemoradiation features in prediction of treatment response. Absolute diffusion coefficient (ADC) had the highest accuracy but, when combined with texture analysis, classification performance increased (accuracy = 82.8%). When T2-weighted texture features were evaluated independently, their best combination of pre-chemoradiation indices was equivalent in accuracy (81.8%)	An accurate assessment of response to chemoradiation in head and neck cancer patients could potentially be predicted from ADC parameters combined with texture analysis of T2-weighted imaging	2	1

Classification of progression free survival with nasopharyngeal carcinoma tumors	Farhidzadeh H, Kim JY, Scott JG, Goldgof DB, Hall LO, Harrison LB	3/24/2016 (ePub)	25	Nasopharynx	Contrast-enhanced T1-weighted	Manual and autosegmentation	No	Not specified	PFS (dichotomized)	Texture features derived from highly-enhancing signal intensity subregions classified PFS with 80% accuracy (AUC: 0.60). Texture features derived from weakly-enhancing subregions classified PFS with 76% accuracy (AUC: 0.76)	Intratumoral textural variations obtained through radiomics analyses can provide a "novel metric" to predict prognosis and assist clinicians in the design of individualized treatment regimens	1	1

A Magnetic Resonance Imaging-based approach to quantify radiation-induced normal tissue injuries applied to trismus in head and neck cancer	Thor M, Tyagi N, Hatzoglou V, Apte A, Saleh Z, Riaz N, Lee NY, Deasy JO	3/25/2017 (ePub); 1/2017 (Print)	20	Head and neck (sites not specified)	Contrast-enhanced T1-weighted	Manual	No	A Computational Environment for Radiotherapy Research	Radiation-induced trismus	Univariate statistical associations were derived. Mean dose to masseter (M), mean dose to medial pterygoid (MP), and Haralick correlation [gray-level co-occurrence matrix (GLCM)] of MP demonstrated the best discriminative ability in characterizing radiation-induced trismus (AUC: 0.85, 0.77, and 0.78, respectively)	An interplay between dose to M and MP as well as GLCM of MP suggests a possible relationship relevant to the etiology of radiation-induced trismus	1	1

Magnetic resonance imaging sequences also varied, with T1-weighted, T2-weighted, and contrast-enhanced T1-weighted scans representing the most commonly used sequences. Only four studies (25%) derived texture features from a single MRI sequence. Thor et al. (45) extracted 24 textures, containing first- and second-order features, from T1-weighted post-contrast images to quantify radiation-induced trismus. Brown et al. (36) investigated whether 21 texture features from a set of 300 DWI MRI parameters could reliably predict histopathological classification of thyroid tumors. Jansen et al. (40) generated pharmokinetic maps from DCE MRI images, applying texture measures of energy and homogeneity to determine associations with treatment response in oropharyngeal cancer patients.

Region of interest segmentation methods were less variable: Manual segmentation by trained experts alone (62.5%) composed the majority of studies. This was followed by combined manual and autosegmentation (31.25%), with one segmentation method unspecified (6.25%). One study investigated the classification performance of an autosegmentation method. Fruehwald-Pallamar et al. (38) leveraged a three-step strategy: Atlas-based registration, support vector machine (SVM) feature training, and parotid volume segmentation using trained feature SVM. For validation, reliability of the autosegmentation method was compared with trained physician contours using a Dice overlap ratio.

Most studies (62.5%) clarified image pre-processing steps before feature extraction. Preferred software for feature extraction included Matlab (37.5%) (MathWorks, Natick, MA, USA) and MaZda (25%) (Institute of Electronics, Technical University of Lodz, Poland). Feature pre-processing and model selection methods are discussed in the “Checklist scores” section of this manuscript.

Final radiomic signatures ranged from inclusion of 2 to 279 features. The upper limit reflects the choice of one study to maintain their initially derived feature set, which was not reduced in dimensionality. Meyer et al. (41) generated 279 features from T1-weighted and T2-weighted images corresponding to the following categories: gray-level co-occurrence matrix (GLCM), gray-level histogram, gray-level run-length matrix, gray-level absolute gradient, auto-regressive model, and wavelet transform. They then compared the derived T1- or T2-weighted features to cellular density, presence of Ki-67 antigen, or p53 index histopathology in 12 thyroid cancer patients.

Reports of radiomic model performance were typically positive (93.75%). However, Fruehwald-Pallamar et al. (39) concluded texture analysis was not practical across multiple MRI protocols, scanners, and vendors. Table 1 lists the statistical findings specific to radiomic model performance of each study. Linear discriminant analysis (LDA) was the most commonly identified classification method, with four studies (25%) leveraging LDA to combine or reduce feature subsets. Likewise, four studies (25%) investigating progression outcomes in NPC patients utilized least absolute shrinking and Lasso methods to select significantly associated features for inclusion in final models. Only seven studies (44%) completely reported the predictive performance of their final model, in terms of their validation strategies, parameter estimates, and confidence intervals (CIs).

Analyzed endpoints ranged from segmentation and histopathological classification categories (44%) to prognostic or predictive biomarkers (56%). Among studies evaluating segmentation or classification, analyzed endpoints included: Histopathological classification (85.7%) and segmentation accuracy (14.3%). For studies assessing prognostic and predictive biomarkers, endpoints included: treatment response (33.3%), progression-free survival (PFS) (22.2%), progression dichotomized (22.2%), prognostic performance of predicting local or distant treatment failure (11.1%), and presence of radiation-induced trismus (11.1%).

All six NPC studies investigated prognostic or predictive biomarkers. Although they contained varying sample sizes (100–118), four studies (42, 47–49) selected from the same number of extracted radiomic features (970), subsequently constructing radiomic signatures from contrast-enhanced T1-weighted or T2-weighted feature categories. Among these studies, three investigated progression (either dichotomized yes/no or analyzed continuously) or a construct of prognostic performance. Liu et al. (2), alternatively investigated treatment response, defined using the Response Evaluation Criteria in Solid Tumors (RECIST). Patients with partial or complete response were considered responders, whereas patients with stable or progressive disease were classified as non-responders. One hundred and twenty six texture parameters were selected from contrast-enhanced T1-weighted, T1-weighted alone, and T2-weighted feature categories, then reduced to 15 features: GLCM, intensity size-zone matrix, and gray-level-gradient co-occurrence matrix. Using two separate selection methods, the remaining NPC study, Farhidzadeh et al. (50), examined the prognostic predictive power of intratumoral features—from either highly or weakly enhancing sub-regions—to classify patients by PFS category.

#### Checklist Scores

Finalized checklist scores are available in our supplementary dataset (Table S2 in Supplementary Material). Liu et al. (2) addressed the highest number of checklist items (TS: 48), followed by Brown et al. (36) and Ramkumar et al. (43) (TS: 45). Of note, all studies scored points for identifying their clinical goals, stating their predictive modeling, defining their target(s) of prediction, describing their sample size, defining the observational units of their response variable(s), interpreting their final model(s), and reporting the clinical implications of their data. By subsection, most study titles (93.75%) identified their reports as introducing a predictive model. Abstracts typically addressed objectives (87.5%), performance metrics in point estimates (87.5%), and practical relevance of study conclusions (87.5%); however, only three abstracts contained information on data sources (18.75%) or framed their performance metrics in terms of CIs (18.75%). Although only six study introductions addressed prediction accuracy of existing models (37.5%), this section contained the highest number of unanimously addressed items (50% of checklist items were unanimously addressed).

Methodology criteria contained the most checklist items [*n* = 32 (58.1%)]. Of the subsections in this category, studies missed the most points for failing to clarify their data (feature) pre-processing: Only seven studies (44%) discussed their data transformation, four (25%) removed outliers, three (18.75%) stated criteria for outlier removal, and one study (6.25%) discussed how missing values were handled. However, missing information in the abstract section, such as data sources, was eventually addressed in study methods (75%). Other common omissions included failures to specify model selection strategies (50% addressed); to define performance metrics in selecting the best model (37.5%); to explain the practical cost of prediction errors (18.75%); and to identify which independent variables primarily take a single value (6.25%). Subgroup analysis of MS to TS demonstrated collinearity between both scoring sets [Spearman’s ρ = 0.94 (*p* < 0.0001)].

Studies were strong in reporting their predictive performance, but only seven (44%) completely addressed their metrics in terms of validation strategies, parameter estimates, and CIs. A list of measured outcomes reported in each study is available in our supplementary material (Table S4). In addition, just one study (6.25%), Fruehwald-Pallamar et al. (38), compared their strategy with existing models in the literature using CIs. As for their conclusions, studies consistently failed to demonstrate whether sufficient data were available to fit their respective models (25%). However, most addressed potential bias (62.5%) as well as generalizability (68.75%) of their data.

### Synthesized Findings of Ongoing Trials

Ongoing trials (51–54) (Table 2) estimate completion dates between June 2018 and December 2019 with one end-date unknown (25%). Three studies did not indicate a specific MRI sequence for feature extraction (75%). In addition, three studies will evaluate multiple head and neck sub-sites (75%). Two studies will prospectively evaluate data (50%), one study will be a case series (25%), and one study did not specify its design (25%). All studies will evaluate prognostic or predictive endpoints and, in addition, one study will evaluate a decision support system as its primary endpoint (25%). No preliminary data are available for any of the ongoing studies.

**Table 2:** Magnetic resonance imaging (MRI) radiomics in HNC: ongoing trials

Article title	Article authors	Publication date	Number of patients	Head and neck sub-site	MRI modality and/or sequence used for radiomics analysis	ROI segmentation method	Image pre-processing: yes/no	Feature extraction software	Analyzed endpoint	Statistical findings: radiomic model performance or conclusions	Successful search terms used [1 = Radiomic(s), 2 = MRI texture analysis, 3 = texture analysis, 4 = head and neck, 5 = magnetic resonance imaging texture analysis]	Databases (1 = PubMed, 2 = EMBASE, 3 = NIH, 4 = ClinicalTrials.Gov, 5 = ChiCTR)
Big data and models for personalized head and neck cancer decision support (BD2DECIDE)	Poli T, Schcekenback K, Schipper J, Colter L, Licitra L, Gatta G, Favales F, Trama A, De Cecco L, Silini EM, Maglietta G, Caminiti C, Iambin P, Hoebers F, Berlanga A	Estimated study completion date: 4/2019	Prospective arm: 450, Retrospective: 1000	Head and neck (Oral cavity, oropharynx, larynx, hypopharynx)	T1-weighted; T2-weighted; Computed Tomography (CT)	Not specified	Not specified	Not specified	Validation of decision support system; secondary outcomes include improved quality of life and assessment of survival time	N/A	1	4

Predictors of normal tissue response from the microenvironment in radiotherapy for prostate and head-and-neck cancer (MICROLEARNER)	Valdagni R, Orlandi E, Bedini N, Cecco LD, Zaffaroni N, Rancati T	Estimated study completion date: 12/31/2019	Prospective clinical trial population: 130 prostate, 130 HNC; prospective validation population: 70 prostate, 70 HNC	Prostate; Head and neck (oral cavity, pharynx, larynx, paranasal sinuses and nasal cavity, salivary glands)	MRI (not specified)	Not specified	Not specified	Not specified	Acute toxicity <90 days after Rt; secondary outcomes include late toxicity	N/A	1	4

Radiomics features for prediction of effect of local advanced nasopharyngeal carcinoma based on CT or MRI pre-chemoradiotherapy—a prospective cohort study	Su T-S	Estimated study completion date: TBD	Case series of 200	Nasopharynx	CT or MRI (not specified)	Not specified	Not specified	Not specified	Overall survival (OS), secondary outcomes include local-control rate and progression-free survival (PFS)	N/A	1	5

Personalized postoperative radiochemotherapy in patients with head and neck cancer	Zips DA	Estimated study completion date: 6/2018	Not specified	Head and neck (oropharynx and hypopharynx)	Positron Emission Tomography (PET), MRI (not specified)	Not specified	Not specified	Not specified	PFS; secondary outcomes—disease free survival, OS, development of a multi-parametric decision support system	N/A	1	4

## Discussion

### Summary of Main Findings

Our review represents the first attempt to summarize MRI radiomics research in HNC patients. Each completed study was evaluated using checklists generated from Luo et al. (1) EQUATOR methodology reporting guidelines: Individually scored, then collectively assessed for quality. Overall, our results indicate significant heterogeneity in study design, with limited consensus on a preferred radiomic signature. Thus, despite addressing reporting guidelines, included studies still demonstrate poor standardization. Such deficits may limit their generalizability and eventual use as clinical-decision support systems. However, this comprehensive review may improve comparison of data across study methodologies and structure similar analyses in other cancer sites.

#### Addressing Study Design

Several factors contribute to the lack of standardization across MRI radiomic studies in HNC patients. Variations follow the typical radiomics workflow: Patient populations (or head and neck sub-sites), image acquisition and pre-processing (MRI modalities), ROI segmentation methods, image pre-processing and feature extraction, feature selection, statistical modeling, and analyzed endpoints.

#### Head and Neck Sub-Sites

In our analysis, there was not a single head and neck sub-site representing a majority of all studies. However, the nasopharynx (37.5%) was the most commonly researched site. Diversity in head and neck sub-sites is not a unique characteristic of MRI radiomic studies, as research using CT radiomics has demonstrated a similar range of investigated patient populations (14). However, the high percentage of NPC studies may reflect the frequent use of MRI in their standard of care (55, 56).

In all six NPC studies, radiomic signatures demonstrated predictive potential. Of the feature categories included in their final radiomic signatures, GLCM was the only shared feature category between studies. This is consistent with NPC radiomic studies using other imaging modalities: Lu et al. (57) analyzed 88 texture features from FDG/PET-CT scans of 40 NPC patients, calculating the robustness of selected parameters in segmentation and discretization. Five GLCM properties (SumEntropy, Entropy, DifEntropy, Homogeneity1, and Homogeneity2) significantly demonstrated robustness at an intraclass coefficient constant ≥0.8 for seven segmentation methods and five discretization bin sizes.

Magnetic resonance imaging radiomics is not limited to studies of tumors alone. Radiomic signatures can predict RT-related toxicities in normal tissues, such as radiation-induced trismus (45), or they can be designed to autosegment parotid glands post-RT (46). Future studies should investigate whether radiomic features could predict the effects of RT-related toxicities on quality of life or if changes in corresponding critical organ volumes, such as structures involved in the swallowing mechanism, can be estimated.

#### MRI Modalities

Magnetic resonance imaging sequence preferences varied among studies, which is not uncommon to radiomics research in other cancer sites (58). Multiparametric approaches may reduce the risk of bias from features extracted from one sequence alone (49). However, since Brown et al. (36) and Jansen et al. (40) evaluated physiologic parameters, it is reasonable that additional MRI sequences would not adequately address their respective hypotheses. For example, Jansen et al. (40) selected DCE MRI for its ability to incorporate pharmacokinetic modeling. Before their study, DCE MRI parametric maps exhibited high image coherence among a tumor response group of limb sarcoma patients (59). Brown et al. (36) chose DWI MRI to improve its accuracy in stratification of thyroid nodules, a utility proven in feasibility studies (60, 61).

Other than sequence selection, MRI modalities may differ in their scanner properties, which would affect the reproducibility of images and, in turn, the texture features derived from them. To investigate whether texture-based signatures could appropriately classify head and neck masses across centers, Fruehwald-Pallamar et al. (39) recruited five MRI scanners from multiple manufacturers—each with varying field strengths, sequences, and acquisition parameters. The objective was to test whether texture analysis could be reliably reproduced in a “real world” clinical scenario. Although the authors ultimately could not recommend texture analysis for routine practice, certain texture features maintained discriminatory significance—particularly those derived from short tau inversion recovery and T2-weighted sequences. However, a review of study methodology revealed omissions in model selection strategy, and their overall checklist score was below the median (TS: 37). Another issue was their intentionally diverse study population. Even though the sample consisted of 100 patients, the sub-sites were heterogeneous, with an unequal distribution of tumors among seven categories of benign masses and five categories of malignant masses. Thus, it is difficult to draw conclusions on radiomic signatures off this study alone.

Although the Quantitative Imaging Biomarkers Alliance (QIBA) continues to develop protocols for optimizing acquisition parameters, a technically confirmed profile for MRI radiomics does not exist. Yet, functional magnetic resonance imaging, DWI MRI, DCE MRI, and magnetic resonance elastography imaging biomarker profiles are currently in progress. The QIBA profile on DWI MRI (62), for example, specifies quality analysis (QA) of image acquisition and review of acquired data in brain, liver, and prostate studies. QIBA designed DWI MRI phantoms to streamline calculations of absolute diffusion coefficient (ADC) parametric maps and bias estimates, signal-to-noise ratios, as well as ADC spatial and *b*-value dependences. Extension of this protocol to DWI MRI radiomic studies in thyroid cancer could thus standardize ADC ROI assessment.

#### ROI Segmentation Methods

Once useable images are generated, ROIs must be segmented to assign volumes for feature derivation. Similar to other processes in the radiomics workflow, segmentation methods vary in their approach and design. Volumes are typically delineated either by manual contours, which can be laborious and time-consuming, or through autosegmenting machine-learning algorithms (63). Although the latter may present a new opportunity for standardized segmentation methods, challenges persist related to the complex anatomy of the head and neck sub-site, optimization of patient-based atlases, and SVM training characteristics (46). Further still, such methods may pale in comparison to recent advances in deep learning, where autosegmentation of myocardial volumes has already been accomplished on cardiac MRI (64). For studies leveraging one segmentation method alone, QA must be specified to limit ROI variation error. Example QA strategies include utilizing multiple experts to review volumes or statistically validating segmentation methods, as Fruehwald-Pallamar et al. (38) optimally demonstrated.

#### Image Pre-Processing and Feature Extraction

Before feature extraction, image quality should be ensured through pre-processing steps. To mitigate noise, which may confound raw imaging data, filters can be applied. Filter choice is dependent on acquisition parameters of imaging modalities, which necessitates standardization of preceding steps. Other obstacles to image pre-processing include diverse resampling schemes, varying computational definitions, motion artifacts, tumor size, and intratumoral heterogeneity, all of which need to be accounted for in study methodology (65, 66). As an example, Liu et al. (37) not only specified the standardization of their image acquisition parameters but also detailed their protocol for normalizing variations in image gray-level ranges.

Feature extraction ultimately depends on choice in software as well as characteristics of the features themselves. Radiomics features can be categorized by statistical output, where each subsequent ordinal group represents a higher complexity of voxel-based analysis. For example, first-order characteristics (e.g., ADC) are spatially independent descriptors of voxel distribution. Second-order characteristics, often equated with textural features, describe spatial relationships between two neighboring voxels (12). Often, however, studies do not explicitly characterize their extracted feature set, a major limitation to research reproducibility. At the minimum, the included studies in this review extracted spatially dependent features to investigate their endpoints.

#### Feature Selection

Each study developed a unique radiomic signature, which demonstrates both the strengths and weaknesses of “big data” research. Strengths include the volume of potentially useful quantitative information and flexibility of radiomic applications, but reproducibility and reliability of measured outcomes remain a concern (65). Thus, comparison of all selected features between studies is not entirely feasible. Although radiomic signatures contained similar categories of features, *diverse* parent feature samples derived from *diverse* MRI sequences with *their own diverse* scanner properties, signify the level of input and output variation inherent to these studies.

While most included studies detailed selection of extracted radiomic features, Meyer et al. (41) did not reduce their initially derived feature set. Direct and inverse correlations between specified features and classification parameters were discovered, but this presents a challenge to rationalize statistically. Potentially spurious associations (e.g., false positives) are inadequately addressed, which reflects the issues (e.g., approaches to data cleaning and transformation) identified collectively in our checklist. Future studies should clearly justify handling of missing values as well as terms and conditions for outlier removal. As checklist scores indicate, this remains an unaddressed issue.

Investigating the stability of MRI radiomic signatures could also identify necessary tweaks to the system. For instance, a feature selection method based on established stability criteria may help guide standardization of radiomic signatures (65). In soft tissue sarcomas, DWI MRI radiomic features derived from ADC maps were shown to maintain relevance across geometric transformations of ROIs (67). In recurrent GBM, test-retest reproducibility of 158 second-order radiomic features revealed 74% stability (68). Similarly, Liu et al. (2) only incorporated reproducible textural parameters in their final radiomic signature. They used a concordance correlation coefficient ≥0.9 to initially select features that maintained stability across different multi-observer ROI iterations of the same NPC patient. Outside of validation datasets, however, similar approaches are lacking in HNC studies.

#### Statistical Modeling

Discussed in previous reviews, a final radiomic signature is constrained by statistical analysis (9, 69, 70). When building predictive models, a set of candidate models should be reduced to the most appropriate classifier, defined by performance metrics of a specific selection strategy (e.g., *k*-fold validation) (1, 66). Otherwise, a concern may be the adoption of dimensionality-reduction techniques solely to limit over-fitting of data. A combined feature extraction and statistical learning platform, built for radiomic challenges, would quell concerns about optimization of radiomic models. Until then, the aforementioned barriers persist across imaging modalities, with limited research focused exclusively on MRI radiomic applications (65).

#### Analyzed Endpoints

Choice of analyzed endpoint guides investigators through their specific radiomics pipeline. Thus, this adds another layer of complexity to selection, extraction, and modeling of features. To objectively predict outcomes, then, automating the above steps may preclude confounded associations. In their prospective MRI radiomic analysis of head and neck tumor p53 classification, for example, Dang et al. (37) used separate software for feature quantification and selection to identify best candidate predictors. Textural features can be biased by imbalances in events or classification parameters, particularly for prediction of rare outcomes. Statistical sampling techniques to enhance prediction accuracy should be implemented for unbalanced datasets.

In their 2016 review of HNC radiomics, Wong et al. (14) identified four of the included studies in our cohort, with three (75%) investigating classification schemes and just one (25%) analyzing prognostic or predictive biomarkers. At the time, CT radiomics research in HNC concentrated on the latter category (14). Discovered through our search strategy, abstracts from conference proceedings (Table S3 in Supplementary Material) all focused on prognostic endpoints in NPC patients (71–73). Thus, perhaps, MRI radiomic studies in HNC are trending toward these outcome measures.

#### Checklist Scores

Studies with the highest overall scores [e.g., Liu et al. (37) (TS: 48)] addressed more of the methodology reporting guidelines than studies with lower scores (Spearman’s ρ = 0.94), which reflects areas of improvement for subsequent work. For example, Liu et al. (2) (MS: 30), were awarded points across the category except for one item (stating how missing values were handled). In addition to an internal 10-fold cross-validation strategy, the study externally validated their findings in an independent sample of 11 patients. They were also the only study to address each item in the “Build the predictive model” subsection. Their manuscript’s discussion received points for every item in the “limitations” subsection; in particular, the authors demonstrated sufficient data available for fitting of their models (neglected in 75% of studies).

Likewise, Ramkumar et al. (43) addressed methodology items commonly missing in other studies. For instance, the authors explained possible prediction errors of texture analysis in distinguishing sinonasal squamous cell carcinoma from inverted papilloma. Similarly, they addressed multiple items in the data pre-processing subsection including data cleaning (e.g., feature reduction) and data transformation. The study meticulously described organization and selection of features, *via* a principal component analysis, as well as the metrics in building their final model. Although not technically an external validation set, the addition of a neuroradiologist review to an internal leave-one-out cross-validation assess buffered the strength of their classification accuracy.

### Limitations

The review does present some notable limitations. A literature search with a known end-date may miss studies published in the interim; this is a limitation of any systematic review. Since MRI radiomics is a field still in its infancy, with a nomenclature not fully standardized, search keywords based on existing literature may not detect all eligible works inclusively. Specifically, keywords containing “texture analysis” may not encompass the breadth of radiomic investigations. To address this, we combed references of each included manuscript. Yet, we are aware of the challenges and risk of bias in selecting potential studies for inclusion and presenting a complete summary of a burgeoning research topic.

Although our checklist was constructed from established guidelines (1), the scoring system required multiple revisions to fairly assess the included studies. As the guidelines were not intended to be quantitative measurements, our group met frequently to weight each item. In addition, we removed guidelines which were difficult to interpret among all authors. Finally, we cannot predict whether the original authors of the guidelines would have constructed the same checklist. We can, however, attest to its quality, given its review by multiple expert radiation oncologists trained in radiomic analyses.

### Conclusion

Magnetic resonance imaging radiomic studies in HNC lack standardization of study design, which practically limits their clinical relevance. Nonetheless, radiomic applications have demonstrated predictive potential in classification schemes and prognostic biomarker identification. Our quantitative scoring system may encourage routine study assessment, perhaps ensuring better data moving forward.

As our collation of the available HNC evidence indicates, MRI radiomics is an evolving field of study. Thus, we suggest several steps for streamlining future investigations. At our institution, novel radiomic-specific MRI phantoms are currently in development and may quantify the effects of inter-scanner variability on radiomic feature generation (70). Understanding the interplay between these processes will hopefully enhance data output. Regarding extraction and selection of features, the imaging biomarker standardisation initiative continues to derive testable categories (74). However, feature stability assessments in MRI are still pending. Analysis should be conducted using readily available software with sufficient flexibility across statistical platforms. Reports of finalized results should follow Luo et al. (1) EQUATOR methodology reporting guidelines.

To cross-validate radiomic signatures externally, tests should be performed on public patient datasets (e.g., The Cancer Imaging Archive). To this end, an upcoming multi-site collaboration between MDACC and other academic cancer centers will generate a repository of patient data in Digital Imaging and Communications in Medicine format, as part of our LAMBDA-[RAD]^2^-HN initiative: a Large-scale Image Aggregation for Machine-Learning/Big Data Applications in Radiomics/Radiotherapy for Head and Neck Cancer. This working group aims to provide an open-access library of curated “big data,” rigorously maintained and routinely assessed for quality (75). Therefore, subsequent efforts to standardize MRI radiomics in HNC would share a reliable data pool.

## Author Contributions

Study designed by all authors. Literature search performed by AJ, TL, and SV. Data extraction completed by AJ and TL. Quality check completed by HE. Data synthesis of selected studies completed by AJ, TL, and HE. All tables formatted by AJ. Checklist designed by TL. Checklist structure revised by AJ and HE. Checklist scores for each study calculated by AJ and TL. Discrepancies between author checklist scores resolved by AJ, TL, and HE. Consort diagram designed by TL. Abstract drafted by SV, HE, and AJ. Cover letter and manuscript drafted by AJ. Abstract, cover letter, and manuscript reviewed and edited by SV, TL, HE, AM, PY, and CF.

## Conflict of Interest Statement

The authors declare that the research was conducted in the absence of any commercial or financial relationships that could be construed as a potential conflict of interest.

## Supplementary Material

Table S1 Blank checklist.

Table S2 Finalized checklist scores.

Table S3 MRI radiomics in HNC: abstracts only.

Table S4 Reports of measured outcomes.

Table S5 Search strategy.

## Abbreviations

ADC, absolute diffusion coefficient; ARM, auto-regressive model; CCC, concordance correlation coefficient; ChiCTR, Chinese Clinical Trial Registry; CI, confidence interval; CT, computed tomography; DCE, dynamic contrast-enhanced; DICOM, digital imaging and communications in medicine; DWI, diffusion-weighted imaging; EQUATOR, Enhancing the Quality and Transparency of Health Research; FDG/PET, fludeoxyglucose-positron emission tomography; fMRI, functional magnetic resonance imaging; GBM, glioblastoma; GLAG, gray-level absolute gradient; GLCM, gray-level co-occurrence matrix; GLGCM, gray-level gradient co-occurrence matrix; GLH, gray-level histogram; GLRLM, gray-level run-length matrix; HNC, head and neck cancer; HU, Hounsfield unit; IBSI, image biomarker standardisation initiative; ICC, intraclass coefficient constant; IP, inverted papilloma; LAMBDA-[RAD]^2^-HN initiative, a Large-scale Image Aggregation for Machine-Learning/Big Data Applications in Radiomics/Radiotherapy for Head and Neck Cancer; LDA, linear discriminant analysis; MDACC, MD Anderson Cancer Center; MRE, magnetic resonance elastography; MRI, magnetic resonance imaging; MS, methodology score; NCBI, National Center for Biotechnology Information; NIH RePORTER, National Institute of Health Research Portfolio Online Reporting Tool; NPC, nasopharyngeal cancer; NSCLC, non-small cell lung cancer; OPC, oropharyngeal cancer; PCA, principal component analysis; PFS, progression-free survival; PRISMA, Preferred Reporting Items for Systematic Reviews and Meta-Analyses; QA, quality analysis; QIBA, Quantitative Imaging Biomarkers Alliance; QoL, quality of life; RECIST, Response Evaluation Criteria in Solid Tumors; ROI, region of interest; RT, radiotherapy; SCC, squamous cell carcinoma; SI, signal intensity; SNR, signal-to-noise ratio; STIR, short tau inversion recovery; SVM, support vector machine; TCIA, The Cancer Imaging Archive; TS, total score; WT, wavelet transform.
