# Supplementary material for: Exploring Applications of Radiomics in Magnetic Resonance Imaging of Head and Neck Cancer: A Systematic Review
Source: Front Oncol. 2018 May 14;8:131. doi: 10.3389/fonc.2018.00131 (PMC5960677; doi:10.3389/fonc.2018.00131)
Supplement: Table S5 — Search strategy. [file data_sheet_5.docx]

**Supplementary Material 5. Search Strategy**

#1: “head and neck” [Text Word]

#2: “head and neck” [Text Word] AND “neoplasms” [MeSH Terms]

#3: “head and neck neoplasms” [MeSH Terms]

#4: “magnetic resonance imaging” [MeSH Terms] OR “MRI” [Text Word]

#5: “texture analysis” [Text Word]

#6: “magnetic resonance imaging” [MeSH Terms] AND “texture analysis” [Text Word]

#7: “MRI” [Text Word] AND “texture analysis” [Text Word]

#8: “radiomic” [Text Word] OR “radiomics” [Text Word]
